# Polish Natural Bee Honeys Are Anti-Proliferative and Anti-Metastatic Agents in Human *Glioblastoma multiforme* U87MG Cell Line

**DOI:** 10.1371/journal.pone.0090533

**Published:** 2014-03-04

**Authors:** Justyna Moskwa, Maria H. Borawska, Renata Markiewicz-Zukowska, Anna Puscion-Jakubik, Sylwia K. Naliwajko, Katarzyna Socha, Jolanta Soroczynska

**Affiliations:** Department of Bromatology, Medical University of Bialystok, Bialystok, Poland; Duke University Medical Center, United States of America

## Abstract

Honey has been used as food and a traditional medicament since ancient times. However, recently many scientists have been concentrating on the anti-oxidant, anti-proliferative, anti-inflammatory and other properties of honey. In this study, we investigated for the first time an anticancer effect of different honeys from Poland on tumor cell line - *glioblastoma multiforme* U87MG. Anti-proliferative activity of honeys and its interferences with temozolomide were determined by a cytotoxicity test and DNA binding by [H^3^]-thymidine incorporation. A gelatin zymography was used to conduct an evaluation of metalloproteinases (MMP-2 and MMP-9) expression in U87MG treatment with honey samples. The honeys were previously tested qualitatively (diastase activity, total phenolic content, lead and cadmium content). The data demonstrated that the examined honeys have a potent anti-proliferative effect on U87MG cell line in a time- and dose-dependent manner, being effective at concentrations as low as 0.5% (multifloral light honey - viability 53% after 72 h of incubation). We observed that after 48 h, combining honey with temozolomide showed a significantly higher inhibitory effect than the samples of honey alone. We observed a strong inhibition of MMP-2 and MMP-9 for the tested honeys (from 20 to 56% and from 5 to 58% compared to control, respectively). Our results suggest that Polish honeys have an anti-proliferative and anti-metastatic effect on U87MG cell line. Therefore, natural bee honey can be considered as a promising adjuvant treatment for brain tumors.

## Introduction

Honey has been known for centuries for its medicinal and health properties. It contains various kinds of phytochemicals with a high phenolic and flavonoid content which contributes to its high antioxidant activity [1], [2], [3]. The quantity of these components varies greatly according to the floral and geographical origin, processing, handling and storage [4]. However, the botanical origin of honey has the greatest influence on its antioxidant activity [5]. Recently, honey has been tested and approved scientifically for its functional and biological properties such as anti-oxidant, anti-inflammatory, anti-bacterial, anti-viral, anti-ulcerous activities and anti-cancer properties [4], [6]–[11].

Honey may provide a basis for the development of novel adjuvant for patients with glioma. Recent studies showed a significant anticancer activity of Malaysian jungle honey on human breast, cervical, oral and osteosarcoma cancer cell lines [12], [13]. Research also showed an anti-proliferative activity of other honeys, like honeys from Manitoba in bladder cancer [9], honeys from Iran in renal cell carcinoma [14], manuka honey in human breast cancer cell, murine melanoma cell [15]. However, until now honey has not been found in studies to show anti-proliferative effects on glioblastoma cancers.

Brain tumors are the second leading cause of cancer related deaths in males up to the age of 39 and in females and children younger than 20 years [16]. *Glioblastoma multiforme* (GBM) is the most aggressive form of brain tumor. Despite standard treatments consisting of surgery, postoperative radiotherapy and temozolomide (TMZ), patient survival remains poor, mainly attributed to tumor inherent radio- and chemoresistance [17], [18]. Despite the continuous improvements in the treatment of GBM during the past decade, these tumors are still associated with a poor prognosis and a rare long-term survival of the patients [19], [20]. Therefore, there is a great need to understand the underlying mechanisms of tumor progression in order to define novel therapeutic targets for GBM.

To the best of our knowledge, this work presents, for the first time, the anticancer effect of honeys from Poland on tumor GBM (U87MG) cell line. The current study is aimed at investigating the anti-proliferative and anti-metastatic effect of honeys on GBM cancer cells. Interaction between honey and TMZ was also estimated. Furthermore, we performed a qualitative analysis of the tested honeys.

## Materials and Methods

### Materials

Four samples of honey were obtained from different apiaries of north-eastern Poland (the Podlasie region) in 2010 year. Honeys have been classified as buckwheat honey (H1), multifloral light honey (H2), willow (S*alix spp.*) honey (H3) and multifloral dark honey (H4) according to declaration by the manufacturer and the palynological analysis. To verify type of honey we also performed the determination of electrical conductivity [21].

### Reagents

Minimal essential medium eagle (MEM) with l-glutamine (292 mg/L), fetal bovine serum (FBS), trypsin-EDTA, penicillin, streptomycin were purchased from PAA Laboratories GmbH (Pasching, Austria); calcium-free phosphate buffered saline (PBS) was from Biomed (Lublin, Poland), *m*ethylthiazolyl diphenyl-tetrazolium bromide (MTT), dimethyl sulfoxide (DMSO) were obtained from Sigma-Aldrich (St. Louis, MO, USA). Acrylamide/bis-acrylamide solution, ammonium persulfate, bovine serum albumin, *brilliant blue R, bromophenol blue, calcium chloride*, glycine, glycerol, TMZ, sodium dodecyl sulfate (SDS), N,N,N'N'-tetramethylethylenediamine (TEMED), trichloroacetic acid, triton X-100, trizma base were obtained from Sigma-Aldrich (St. Louis, MO, USA). The scintillation cocktail was purchased from PerkinElmer (Boston, MA) and methyl-^3^H thymidine from MP Biomedicals, Inc. (Irvine, USA). All other chemicals were of ultrapure grade and obtained from Sigma-Aldrich (St. Louis, MO, USA).

### Diastase activity

The diastase activity of samples was also measured using the Phadebas method according to the International Honey Commission (2009). The absorbance of the sample was measured at 620 nm using deionized water as a reference, and the absorbance of the blank was subtracted from that of the sample solution (ΔA620). The measured absorbance of the solution is directly proportional to the diastase activity of the sample.

### The analysis of the total phenolic content

The total phenolic content (TPC) was measured in water solutions of honey using the Folin–Ciocalteu colorimetric method (FC). The absorbance versus the prepared blank was read at 760 nm using a Cintra 3030 (GBC Scientific Equipment, Australia). The results were expressed as milligrams of gallic acid equivalent (GAE)/100 g of honey. The assays were carried out in triplicates. The data was expressed as mean ± SD and range.

### The estimation of lead (Pb) and cadmium (Cd)

The samples of honey were mineralized using concentrated nitric acid in a closed microwave speedwave system (Berghof, Germany). The concentration of Pb and Cd in honeys was analyzed by an electrothermal atomic absorption spectrometry (ETAAS) on a Z-2000 instrument (Hitachi, Japan). The measurements were performed at 283.3 nm and 228.8 nm for Pb and Cd, respectively; with the Zeeman – effect background correction. The detection limit was 0.78 µg/L and 0.096 µg/L for Pb and Cd, respectively.

A certified reference material – Simulated diet D (Livsmedels Verked National Food Administration, Sweden), was used to test the accuracy of the methods. The Department of Bromatology participates in a quality control program of the estimation of trace elements of the National Institute of Public Health and Institute of Nuclear Chemistry and Technology. The results of the quality control analyses were in agreement with reference values.

### The preparation of honey for the cell line assays

All the samples of the *Apis mellifer*a honey were obtained directly from the beekeepers in the north-east part of Poland (Podlasie region) during spring and summer 2011. We used four types of honey: buckwheat (H1), multifloral light (H2), willow (S*alix spp.*) honey (H3), multifolral dark (H4) for our study. The working concentrations of honey were prepared for each experiment by a serial dilution with a culture medium after which each concentration was filtered using a 0.20 µm sterile filter unit (Sarstedt 83.1826.001). The honey mixture was freshly prepared before being added to cell cultures.

### Cell culture

The studies were performed on human GBM U87MG cell line, which was obtained from the American Type Culture Collection (ATCC, Rockville, MD, USA). The cells were cultured in a humidified incubator at 37°C and 5% CO_2_ atmosphere in MEM supplemented with 10% FBS; 50 U/mL penicillin and 50 mg/mL streptomycin. Sub-confluent cells were detached with trypsin-EDTA solution in PBS and counted in a *Neubauer* hemocytometer. Cells from passage 7 to 9 were used.

### Morphological analysis under light microscopy

For the morphological analysis, a U87MG cell line was seeded in 100-mm dishes at 2.2×10^5^ cells/ml. The cells were treated with 1% and 2.5% concentration of honeys for 24, 48 and 72 h. At the indicated time points, any morphological changes were examined and recorded under a light microscope (Olympus CKX41).

### Cytotoxicity assay

The effects of H1, H2, H3, H4 (0.5%, 1%, 2.5%, 5%, 7.5%) and a combination of honeys (2.5%) with TMZ (20 µM) on the viability of U87MG cell line were studied after 24 h, 48 h and 72 h of treatment. The cells were seeded into 96-well plates in a volume of 200 µl/well at a density of 2×10^4^ cells/well and grown for 22 h at 37°C in a humidified 5% CO_2_ incubator. The cell viability was measured by a quantitative colorimetric assay using MTT, which is based on the conversion of MTT to formazan crystals by mitochondrial dehydrogenases. Water insoluble MTT-formazan crystals formed inside the living cells were dissolved in the DMSO and the absorbance at 570 nm proportional to the number of living cells was measured on a Multimode Plate Reader Victor X3 (PerkinElmer, Singapore). The data was expressed as a percentage of control. Each experiment was performed in triplicate and repeated independently at least three times.

### H^3^-thymidine incorporation

U87MG cells were plated in 24-well plates at 1.5×10^5^ cells/well and exposed to a medium containing DMSO (control), TMZ, H1 (2.5%), H2 (2.5%), H3 (2.5%), H4 (2.5%) or H1–H4 2.5% with TMZ. Cells were cultured for 20, 44 and 68 h prior to the addition of 0.5 µCi of H^3^-thymidine/well. After 4 h of incubation, the medium was removed and the cells were washed twice with cold 0.05 M Tris-HCl and 5% trichloroacetic acid, scrapped and transferred to scintillation cocktail. The level of incorporated H^3^-thymidine was assessed using the Beckman liquid scintillation counter.

### DNA fragmentation assay

Detection of apoptotic cells with fragmented DNA (sub-G_1_ cells) was performed using Nucleocounter NC-3000 system (ChemoMetec, Denmark). U87MG cells were seeded into 6-well plates at density 7×10^5^ cells/well and after 24 h of incubation were treated 5% solutions of different types of honey. After 24 h cells were analyzed according to the instructions of the producer. The Sub-G1 methods relies on the fact, that after DNA fragmentation, small DNA molecules are able to diffuse out of the cells following washing with PBS. Thus after staining with DAPI cells having loss DNA will take up less stain and will appear left of G_1_ peak in a DNA content histogram. The data were analyzed by NucleoView NC-3000 software.

### Enzyme-linked immunosorbent assay (ELISA)

Nuclear extracts, in an amount of 40 µg/well, were used in ELISA. The experiments were performed using DNA-binding ELISAs for activated NF-κB transcription factors (TransAM NF-κB p65/p50/p52 Active Motif) according to the instructions of the manufacturer. This kit is designed specifically for the study of NF-κB subunits. The results are shown as a percentage of control value and are calculated from three independent experiments.

### Gelatin zymography

The gelatin zymography was used to assess the extent of proMMP-2 and proMMP-9 activity. Serum-free media were collected from subconfluent cells treated with 5% H1, H2, H3 and H4 for 24 h, next concentrated 35-fold and mixed with Laemmli sample buffer. After normalizing with the sample of the least total protein, aliquots of the samples were subjected to SDS-PAGE in a 10% gel impregnated with 0.1 mg/mL gelatin. After the electrophoresis, the gels were incubated in 2% Triton X-100 for 30 min at 37°C to remove SDS and in a substrate buffer (50 mM Tris–HCl buffer, pH 7.8, containing 5 mM CaCl_2_) for 20 h at 37°C. Then, the gels were stained with Coomassie briliant blue R250. Gelatinolytic activity was detected as unstained bands on a blue background.

### Statistical analysis

The data was expressed as a mean value ± standard deviation. All data was analyzed using STATISTICA, Version 10.0 using the *Student's t-test* and *Pearson's correlation* to calculate the value significance. *P* values <0.05 were accepted as statistically significant.

## Results

### Diastase activity of honey

Honey contains various kinds of enzymes, one of the most important is α-amylase, which is responsible for the diastase activity. The diastase activity is lower in honeys falsified or stored in improper conditions. International regulations set a minimum value of 8 Schade units for diastase activity [21]. The value of diastase activity in our samples ranged from 5.3 to 17.9 Schade units/g of honey (Table 1). Diastase activity in H3 was lower than the currently applicable standards and other honeys were in the normal range.

**Table 1:** Diastase activity, TPC and content of lead (Pb) and cadmium (Cd) in honey samples.

Code name	Honeys name	Diastase activity [Schade/g]	TPC* ± (SD)^#^ [mgGAE/100 g]	Content of elements [µg/kg]	
				Pb	Cd
**H1**	buckwheat	14.2±0.3	160.7±0.7	19.33±0.9	2.211±0.10
**H2**	multifloral light	17.9±0.2	88.1±2.6	96.60±5.0	5.519±0.23
**H3**	willow *(Salix spp.)*	5.3±0.2	69.4±1.1	21.83±1.1	6.723±0.29
**H4**	multifloral dark	15.1±0.2	133.5±0.5	1.39±0.1	1.661±0.16

^*^TPC - total phenolic content, ^#^SD – standard deviation.

### Total phenolic content in honey

TPC of the different honeys was investigated by the FC assay and the mean values are shown in table 1. According to these results, H1 had the highest TPC values (160.7±0.7 mg GAE/100 g of honey), while the lowest contents were measured in H3 (69.4±1.1 mgGAE/100 g of honey).

### Pb and Cd content in honey

The content of Pb and Cd in honeys was measured by an atomic absorption spectrometry. The mean values of Pb and Cd content in the examined honey samples were from 1.39±0.1 to 96.60±5.0 µg/kg and from 1.661±0.16 to 6.723±0.29 µg/kg, respectively (Table 1). The results were compared to the Polish standards which permit <50 µg/kg Pb and <10 µg/kg Cd [22]. In accordance with the recommendation of FAO/WHO [23], a provisional tolerable monthly intake (PTMI) is more appropriate than provisional tolerable weekly intake (PTWI) and for Cd amounted 25 µg/kg of body weight for adult, i.e., 1500 µg monthly for 60 kg person. The Committee concluded that the PTWI for Pb (25 µg/kg body weight) could no longer be considered health protective and so they withdrew it. The elemental composition of honey samples gives information about environmental pollution. The information is crucial because minerals and trace elements play an important role in many biochemical processes. The result has shown that the Cd concentration in the examined honeys did not exceed the Polish standards. It is worrying that, one of the analyzed honeys (H2) had a very high content of Pb exceeding almost twice the maximum level.

### Morphological analysis under light microscopy

**Figure 1:**
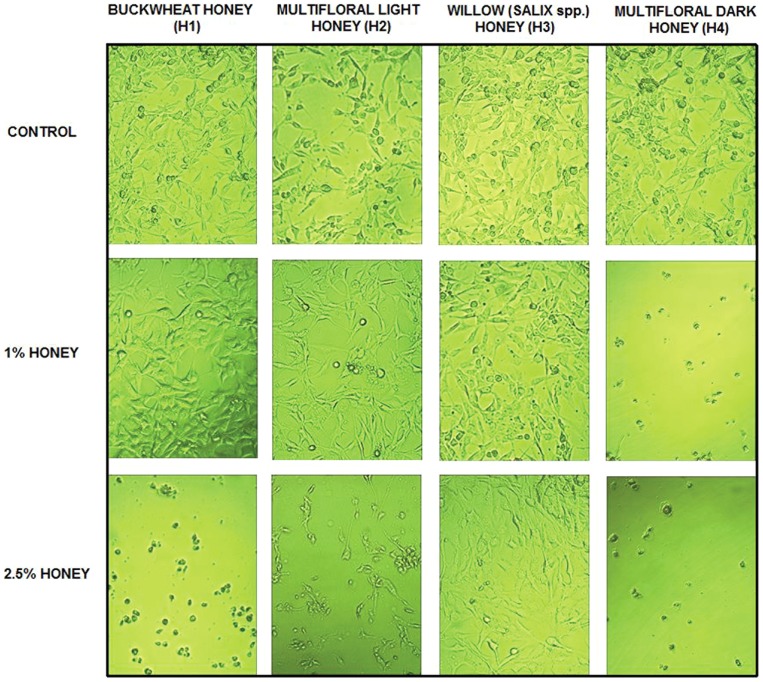
The effect of honeys on morphology of U87MG cell line after 48 h treatment.

In U87MG cell line, cells without honey treatment showed a different branchy and polygonal shape, which is considered as the normal cell growth effect. When the cells were treated with 1% and 2.5% honeys for 48 h the cells were rounded off, shrunk down and showed a decrease in their number (Figure 1).

### MTT cell viability assay

**Figure 2:**
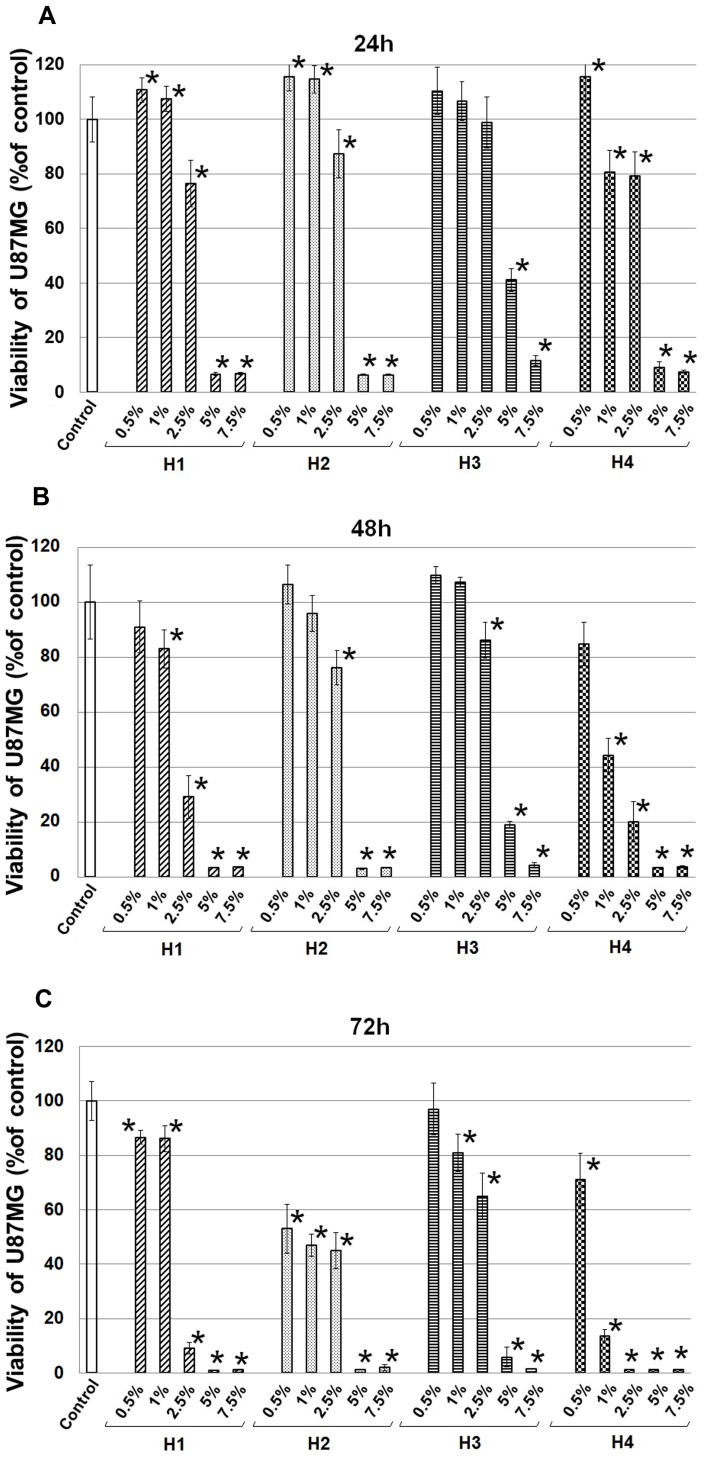
Inhibition of U87MG cell proliferation by polish honeys. U87MG cells were incubated for 24(graph A), 48 h (graph B) or 72 h (graph C) in the presence of the indicated concentrations (range 0.5% to 7.5% w/v) of different (H1–H4) honeys. The results are expressed as percentage viability in treated cell cultures compared to control. Asterisks denote statistically significant differences in the viability of the experimental groups compared to control obtained from the *Student's t-test* (* *p*<0.05).

We examined the cytotoxic effects of honey samples alone and in combination with TMZ on human GBM cell line. We have found a time-dependent - from 24 to 72 h - decrement in a viability of U87MG cells treated with each of the honey samples (Figure 2).

**Figure 3:**
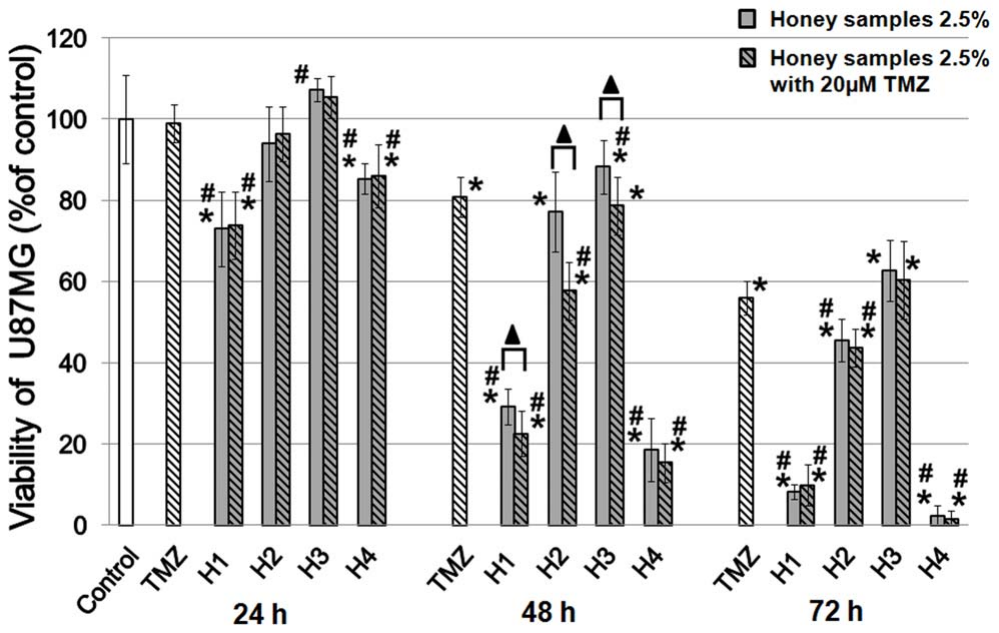
The effect of TMZ, honeys and combination of honey and TMZ on U87MG cell viability. U87MG cells were incubated with the indicated concentration (2.5%) of honeys, alone or in combination with TMZ (20 µM) for 24, 48 and 72 h. Results are expressed as percentage viability in treated cell cultures compared to control. Asterisks denote statistically significant differences obtained from the *Student's t-test*: *p<0.05 vs. control; #*p*<0.05 vs. TMZ; ▴*p*<0.05 honeys alone vs. combination with TMZ.

Treatment with H1 in concentration 2.5% caused significant reduction of viability U87MG cells, compared to control, after 24 h (viability - 76%), 48 h (29%) and 72 h (9%) of incubation; a similar effect was observed after incubation with H4 in concentration 1% (81%, 44%, 13%, respectively). The significant viability decrement of U87MG cells treated with 2.5% H2 was detected after 24 h (viability - 87%); 48 h (76%) and 72 h (45%). Similar effect was observed after 72 h of incubation with 0.5% and 1% H2. H3 showed the weakest reduction of viability compared with other tested honeys (Figure 2). The treatment of U87MG cells with 5% or 7.5% concentrations of honeys – H1, H2 and H4 caused a dramatic reduction of viability. Therefore, for further studies with the combination of honeys/TMZ we chose the most optional concentration of honey at 2.5%. Just as noted in an earlier study, a time-dependent significant reduction of cell viability occurred in comparison with the control. We observed that after 48 h, combining honey with TMZ showed a significantly higher inhibitory effect (viability of 22% - H1; 57% - H2; 78% - H3) than the same samples of honey (H1 - 29%, H2 - 77%, H3 - 88%), while after 24 and 72 h this dependence has not been observed (Figure 3).

### H^3^-thymidine incorporation in U87MG cell line

**Figure 4:**
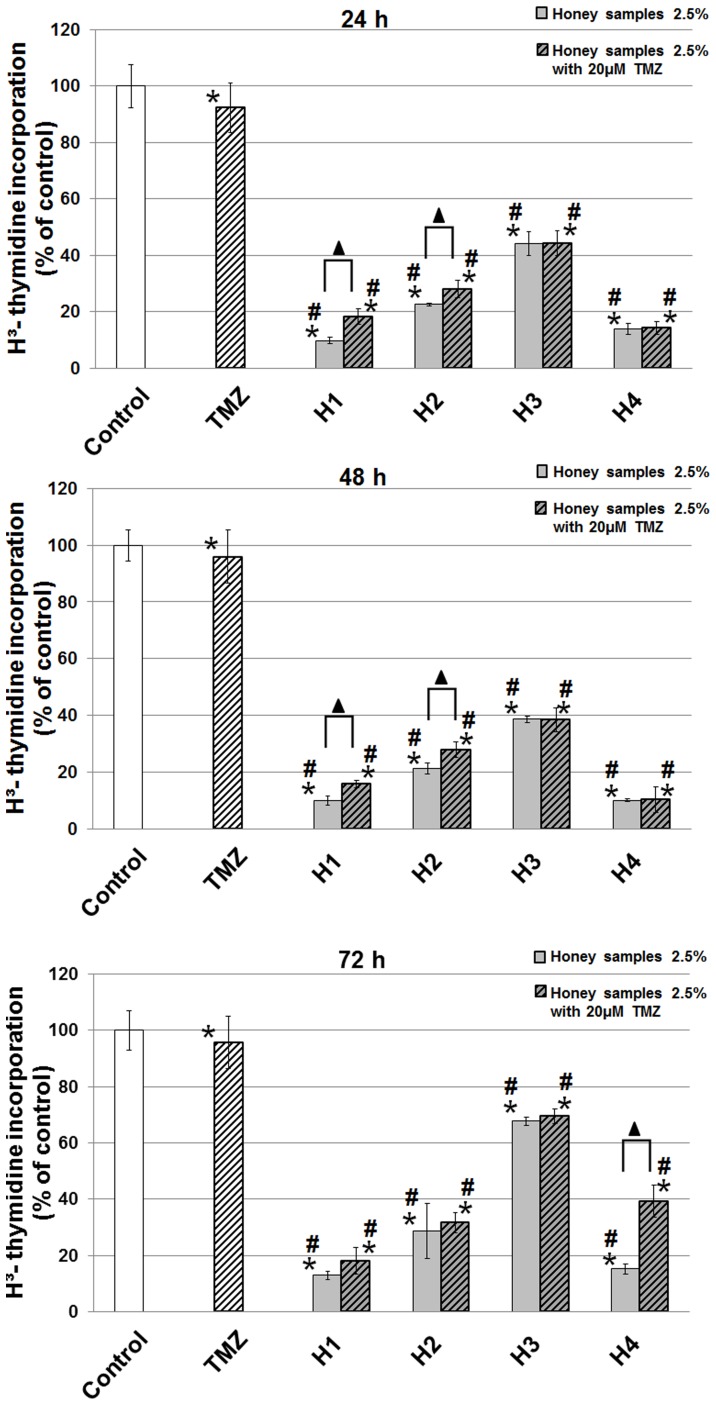
H3-thymidine incorporation after 24, 48 and 72 h of incubation with honey samples alone and together with TMZ. The results are presented as a percentage of control. Significant changes obtained from the *Student's t-test* are indicated at the columns with: *p<0.05 vs. control; #*p*<0.05 vs. TMZ; ▴*p*<0.05 honeys alone vs. combination with TMZ.

In order to check the capability of honeys and the combination of honeys/TMZ and their influence on the DNA synthesis in glioblastoma cells, we evaluated an inclusion of [H^3^]-thymidine for the U87MG cells. All honeys revealed an inhibitory potential on [H^3^]-thymidine incorporation in U87MG cells (Figure 4). The results after 24 and 48 h of incubation were very similar; the strongest effect of decreasing of DNA synthesis versus the control was observed after treatment with H1 (10% after 24, 48 h) and H4 (10% after 24 h and 14% after 48 h). At the same time, TMZ exhibited a minor ability to decrease [H^3^]-thymidine incorporation and a combination of H1 and H2 with TMZ was a little less powerful than H1 and H2 alone. After 72 h of exposure stronger effect was observed for H4 used alone (15% reduction cell division versus the control), than H4 with TMZ (40%). The lowest effect on [H^3^]-thymidine incorporation was observed after 72 h for H3 (68% reduction in DNA synthesis).

### The effect of honey on DNA fragmentation in glioblastoma cell line U87MG

**Figure 5:**
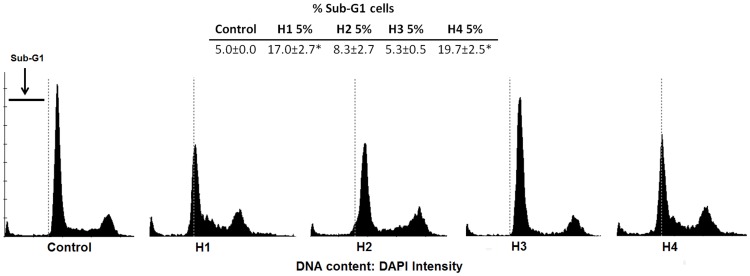
DNA fragmentation in U87MG cells incubated with honey. The percentage of sub-G_1_ phase cells after 24 h treatment with 5% different types of honey. Significant changes obtained from the *Student's t-test* are indicated: *p<0.05 vs. control. The results are mean ±SD from two independent experiment.

During apoptosis, calcium- and magnesium-dependent nucleases are activated which degrade DNA. This means that within DNA there are nicks and double-stand breaks causing fragmentation. After 24 h we observed a significant increase of DNA defragmentation in cells treated with H1 (17.0%) and H4 (19.7%) compared to control (5.0%) (Figure 5).

### The effect of honey on NF-κB activity in glioblastoma cell line U87MG

**Figure 6:**
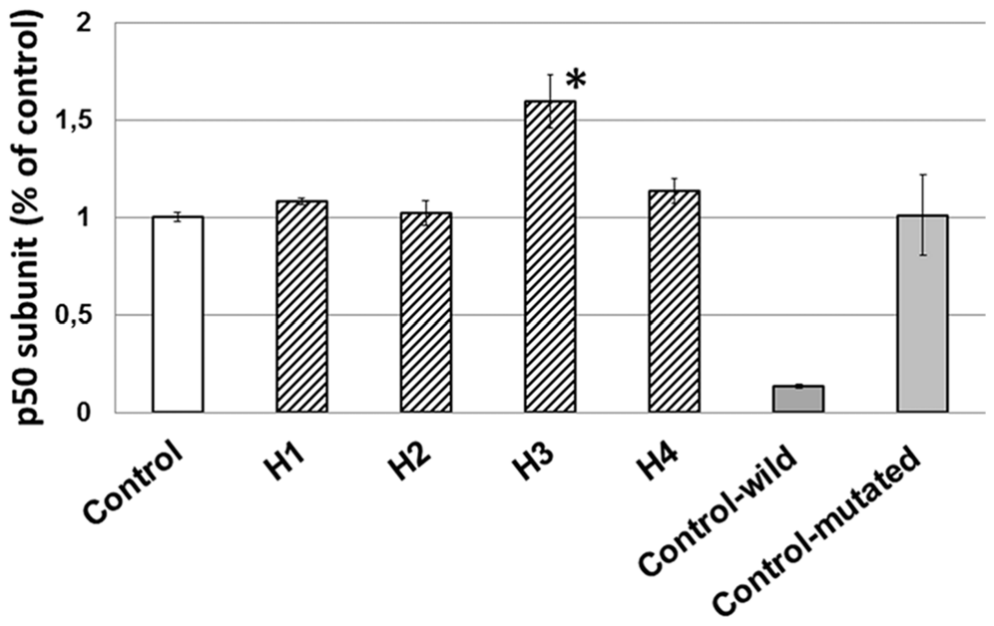
Effect honeys (H1–H4) on NFkB activity in U87MG. The results are presented as a percentage of control. Verification of the ELISA specificity test showed a dramatic decline of p50 signal when a wild-type sequence was used and no decrease when using a mutated sequence. Significant changes obtained from the *Student's t-test* are indicated: *p<0.05 vs. control.

ELISA test was used to measure the concentration of NF-κB p50 subunit. We found that honey did not inhibit the NF-κB activity in U87MG cells (Figure 6). Subunit p50 concentration remained at the level similar to the control after 72 h of incubation with H1, H2, H4. However, it is interesting that H3 caused a significantly increased activity of p50 versus the control. This effect requires further studies to clarify the mechanism.

### The effect of honey on MMP activity in glioblastoma cell line U87MG

**Figure 7:**
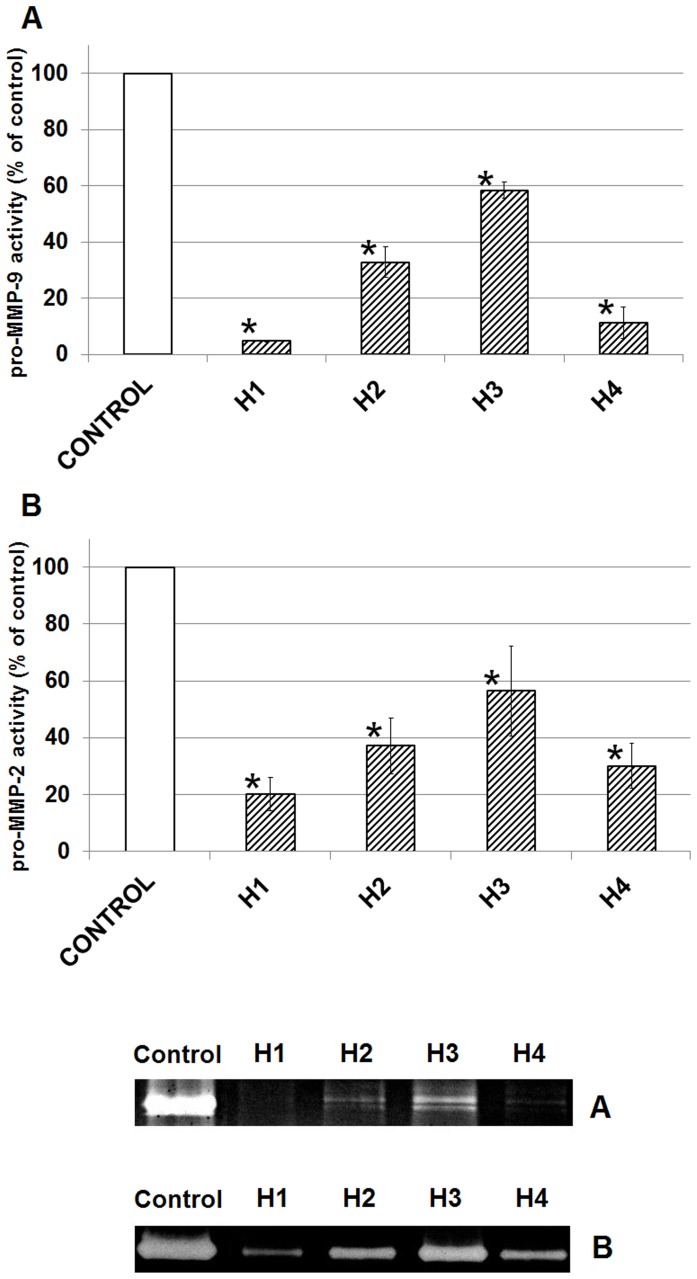
Release and production of MMP9 (A) and MMP2 (B) in cultured U87MG treated with honey. Medium concentrated 35-fold; A-7 µg of protein; B-1 µg of protein. The results are presented as percentage of control. Significant changes obtained from the *Student's t-test* are indicated at the columns with: *p<0.05 vs. control. This pictures is a representative gel of three independent experiments.

The effect of honeys on MMP-2 and MMP-9 activity was investigated using a gelatin zymography assay. Studies have shown that the treatment of U87MG cells with honey samples (5%) for 24 h resulted in a significant decrease in MMP-2 and MMP-9 activity versus the control (Figure 7). We observed the strongest inhibition of MMP-2 and MMP-9 for H1 (about 80% MMP-2 and 95% of MMP-9) and for H4 (about 70% and 89%, respectively). The other two honeys (H2, H3) also inhibited the activity of MMP-2 and MMP-9 strongly.

## Discussion

GBM is the most malignant form of primary astrocytic brain tumors in adults [24]. Patients with GBM usually undergo a treatment which consists of the combinations of surgery, radiotherapy and chemotherapy but have a median survival time of less than one year [25]. TMZ is the most commonly used for patients with GBM and this medicament has proven to improve prognosis [26]. Recently, many patients suffering from cancer or other chronic conditions have been looking for natural products and the use of alternative medicine [27].

Honey has been recognized by many ancient cultures for its healing properties, and recently has been investigated as an anti-cancer agent. The anti-cancer activity of honeys has been analyzed for bladder cancer Manitoba [9], renal cell carcinoma [14], human breast cancer cell and murine melanoma cell [15]. To the best of the authors' knowledge, our research is the first to show the anti-proliferative effects of honey on glioblastoma cell line. In this study, a cytotoxic activity of the examined four samples honey showed a strong cytotoxicity of H4 and H1, and this may be connected with the content of polyphenols in honeys, because the samples H1 and H4 have a much higher TPC than H2 and H3. It should be noted that we have found a significant correlation between TPC and the viability of cells after 24, 48, 72 h of incubation (correlation coefficient r = −0.921; r = −0.907; r = −0.927). This observation confirms the impact of polyphenols on anti-tumor activities. Jaganathan and Mandal [28] suggested that the polyphenols found in honey, like caffeic acid, caffeic acid phenyl ester, chrysin, galangin, quercetin, kaempferol, acacetin, pinocembrin, pinobanksin and apigenin, may be promising pharmacological agents in the treatment of cancer by reviewing their anti-proliferative and molecular mechanisms. According to Galijatovic et al. [29] some bioactive compounds, such as chrysin, found in honey, have been used to prevent cancer, in a similar fashion as anastrozole (a breast cancer drug), and to treat conditions such as anxiety and inflammation. In our study we observed cytotoxic activity of H2 in low concentration (0.5%) after 72 h of incubation. This activity may be associated with a high Pb content. Posser et al., 2007 [30] focused, that Pb may cause a series of effect in C6 glioma cells, including activation of p38MAPK and JNK1/2 and a dose-dependent reduction of cell viability.

TMZ is an imidazotetrazine derivative used in the therapy of malignant gliomas. The mechanism of anticancer action is based on the ability to alkylate DNA, especially at the *O* ^6^ position of guanine [31]. Application of TMZ in the management of high-grade glioma is limited by various resistant mechanisms [31], [32]. A recent study showed that TMZ administered together with one of the natural bee product - propolis [20], [33], [34] enhanced the sensitivity of human brain cancer cells, indicating that the combination of TMZ with that natural bee product may be more effective in glioma therapy than using TMZ alone. In our study the stronger reductions of cell viability and DNA synthesis were observed after treatment with combination honey and TMZ than TMZ alone, however higher effect of honey-TMZ combination compared to honey alone was only detected after 48 h of incubation. According to this observations we can only conclude that TMZ does not inhibit the cytotoxic activity of honey.

Our and other scientific research has shown that honeys decreased the viability of cells and therefore we decided to study the influence of these natural products on DNA synthesis in U87MG cells. The methyl-[H^3^]-thymidine incorporation assay is a widely used, gold standard, method for measuring the inhibition of cell proliferation and has been used successfully to screen and optimize potential new cancer specimens. The results of DNA synthesis after 24, 48, 72 h of exposure to honeys indicate a reduction in U87MG cell proliferation. This fluctuation of DNA synthesis is consistent with the viability of cells in the same time, e.g. the reduction of thymidine incorporation in cells after 72 h is consistent with the reducing of the viability (H1 – 2.5%). This observation is also confirmed by a morphological analysis of cells (Figure 1). One of the indicators of the quality of honey is diastase activity. We find a significantly strong negative correlation between this parameter and DNA synthesis after 24 h (r = −0.784), 48 h (r = −0.763) and 72 h (r = −0.827) of incubation. We found that the intensity of thymidine incorporation depends also on the content of polyphenols in the studied honeys after 24, 48, 72 h treatment of cells (correlation coefficient r = −0.893; r = −0.894; r = −0.842). Polyphenols induce DNA damage by affecting the cell cycle phase (an increase in the number of cells in G2/M and a corresponding decrease in the number of cells in G0/G1) [35]. The data on the influence of bee honey products (especially honey) on DNA synthesis in glioblastoma cells is poor. The inhibition of DNA synthesis was reported for Turkish propolis, which reflected its anti-tumor influence [36]. An ethanolic extract of Brazilian propolis administrated to U937 lymphoma cells caused a reduced cell growth and inhibition of DNA, RNA and protein synthesis [37] and Polish propolis (100 µg/ml) eliminated 90% U87MG cells after 72 h incubation and caused an inhibition of DNA synthesis [34].

The ability to induce tumor cell apoptosis is an important property of a candidate anticancer drug, which discriminates between anticancer drugs and toxic compounds [38]. The changes that we observed in our study (Figure 1) manifested as a loss of cell volume or cell shrinkage can be a morphological hallmark of the programmed cell death process known as apoptosis [39]. Similar changes characteristic of apoptosis in cancer cells have also been described by other authors [40], [13]. Much effort has been directed toward the study of the effect of honey on apoptosis and the understanding the mechanisms of this action. DNA fragmentation is a key apoptotic event. In our study fragmentation DNA was observed in U87MG cells following treatment with buckwheat (H1) and multifloral dark (H4) honey. The accumulation of cells population in Sub-G1 phase may suggest that honey induced apoptosis. An important element in the process of apoptosis in cancer cells is an inhibition of p50 subunit of nuclear transcription factor NF-κB, which is an essential survival factor for many glioblastomas including U87MG cell line [41]. Glioblastomas responded to NF-κB inhibition by reducing the growth rate and an induction of apoptosis [42]. That is why we made enzyme-linked immunosorbent assay, which evaluates the concentration of p50 subunit. Our research has shown that honey samples did not inhibit the NF-κB activity in U87MG cells since nuclear localization of p50. It is interesting that H3 with the highest content of Cd stimulated significantly the activity of p50 (correlation coefficient r = 0.585). Studies on the clarification of the effect on Cd NF-κB activation in the THP-1 human monocytic leukemia cell line show that cadmium activates significantly NF-κB activation [43]. Our unpublished preliminary study using the extract of H3 honey (with a lower content of Cd) showed a different effect-inhibiting activity of p50.

The MMPs are the most important proteolylic enzymes that degrade extracellular matrix to provide an efficient space for glioma to extend, which is essential in the metastasis and an invasion of gliomas [44]–[46]. Our results revealed that the examined honey inhibited MMP-2 and MMP-9 expressions in U87MG cells. It is interesting that honey with a higher content of polyphenols causes a stronger inhibition of the expression of MMP-2 and MMP-9 (correlation coefficient r = −0.828; r = −0.949). Other authors have made a similar observation for different natural products [47], [48]. We focused a moderate negative correlation between diastase activity and expression of MMP-2 (r = −0.652) and MMP-9 (r = −0.681). This may suggest the impact of amylolytic enzymes on the antimetastatic effect of honey. To the best of our knowledge there is no existing data on the effect of honeys on the activity of MMPs in glioma cells. It is interesting that the cadmium content in honey positively correlated with the activity of MMP-2 and MMP-9 (correlation coefficient r = 0.807; r = 0.940). We noticed that H3 inhibits the expression of MMPs less in comparison to other honeys. This may be associated with the highest content of Cd in the honey (Table 1). Probably Cd induces the cleavage of N-cadherin in cells in which γ-secretase is involved [49]. However, the mechanism for honey-induced MMP suppression in glioma cells remains unclear.

## Conclusion

Our results suggest that Polish honeys have a promising antiproliferative effect by cell viability inhibition or decreasing DNA synthesis; furthermore, an antimetastatic effect by inhibiting MMP-2 and MMP-9 on U87MG cell line. We found that the examined products exert different dose- and time- dependent effects on GBM cell lines. Furthermore, it was observed that diastase activity, TPC and Cd contents on the analyzed honeys had impact on their antiproliferative and antimetastatic activity. Therefore, natural bee honey can be considered as a promising adjuvant treatment for brain tumors.
